# Functional and Structural Brain Plasticity in Adult Onset Single-Sided Deafness

**DOI:** 10.3389/fnhum.2018.00474

**Published:** 2018-11-27

**Authors:** Yingying Shang, Leighton B. Hinkley, Chang Cai, Karuna Subramaniam, Yi-Shin Chang, Julia P. Owen, Coleman Garrett, Danielle Mizuiri, Pratik Mukherjee, Srikantan S. Nagarajan, Steven W. Cheung

**Affiliations:** ^1^Department of Otolaryngology-Head and Neck Surgery, University of California, San Francisco, San Francisco, CA, United States; ^2^Department of Otorhinolaryngology, Peking Union Medical College Hospital, Beijing, China; ^3^Department of Radiology and Biomedical Imaging, University of California, San Francisco, San Francisco, CA, United States; ^4^Department of Psychiatry, University of California, San Francisco, San Francisco, CA, United States

**Keywords:** auditory memory, diffusion tensor imaging, magnetoencephalography, plasticity, single-sided deafness

## Abstract

Single-sided deafness (SSD) or profound unilateral hearing loss obligates the only serviceable ear to capture all acoustic information. This loss of binaural function taxes cognitive resources for accurate listening performance, especially under adverse environments or challenging tasks. We hypothesized that adults with SSD would manifest both functional and structural brain plasticity compared to controls with normal binaural hearing. We evaluated functional alterations using magnetoencephalographic imaging (MEGI) of brain activation during performance of a moderately difficult auditory syllable sequence reproduction task and assessed structural integrity using diffusion tensor imaging (DTI). MEGI showed the SSD cohort to have increased induced oscillations in the theta band over the left superior temporal cortex and decreased induced gamma band oscillations over the frontal and parietal cortices between 175 and 475 ms following stimulus onset. DTI showed the SSD cohort to have extensive fractional anisotropy (FA) reduction in both auditory and non-auditory tracts and regions. Overlaying functional and structural changes revealed by the two imaging techniques demonstrated close registration of cortical areas and white matter tracts that expressed brain plasticity. Hence, complete loss of input from one ear in adulthood triggers both functional and structural alterations to dorsal temporal and frontal-parietal areas.

## Introduction

Bilateral deafness or anacusis enforces undesirable auditory isolation and triggers widespread central network plasticity. Those with deafness in both ears compared to controls with normal hearing (NH) have increased activation of auditory association cortex when attending to visual stimuli and performing working memory tasks (Finney et al., 2001, 2003; Lomber et al., 2010; Meredith et al., 2011; Ding et al., 2015) and can develop superior visual perception skills (Dye et al., 2009; Ding et al., 2015). In subjects who acquire bilateral deafness early, the extent of plasticity in auditory cortex appears to be correlated with level of performance enhancement on visuospatialtasks (Ding et al., 2015). Here, we examine whether widespread brain plasticity observed in bilateral deafness may be similarly expressed in unilateral deafness, despite continued access to sound in the only hearing ear.

Unilateral deafness or profound hearing loss is commonly referred to as single-sided deafness (SSD). It is prevalent in adults and children alike and may be congenital or acquired. SSD differs from bilateral deafness in that the only hearing ear can capture sound information for further processing by the central auditory system and related networks. However, an important functional constraint of monaural hearing is loss of panoramic sound access due to the head shadow effect. Some behavioral adaptations to single sensor hearing are deployment of cognitive and attentional resources to monitor the acoustic scene, readiness to orient to sounds of interest by making head or body adjustments, and use of visually guided lip-reading strategies to bolster accuracy. Despite those steps to mitigate challenges imposed by SSD, troublesome clinical consequences include greater difficulty with sound localization, higher signal-to-noise ratio requirement for speech discrimination, and reduced ease of listening (Rigby et al., 1997; Noble and Gatehouse, 2004; Tufarelli et al., 2006; Douglas et al., 2007). Those and other chronic listening difficulties in SSD may stress cognitive networks that subserve attention, working memory, and executive function. Children with SSD exhibit delayed acquisition of oral language and verbal intelligence skills, decreased achievement in school, and impaired executive control when challenged by irrelevant verbal information (Bess and Tharpe, 1984; Brookhouser et al., 1991; Borg et al., 2002; Lieu et al., 2012; Vila and Lieu, 2015). Current treatments for SSD include routing acoustic information to the better ear (Peters et al., 2015), transferring sounds to the contralateral cochlea by bone conduction (Kim et al., 2017), and stimulating the deaf ear by cochlear implantation (Vlastarakos et al., 2014). While the latter intervention reintroduces auditory information through the deaf ear, with promise for restoration of binaural hearing, treatment outcomes remain highly variable (van Zon et al., 2015; Cabral Junior et al., 2016).

Given the wide ranging clinical deficits observed in SSD and lack of reliable predictors to assist with patient selection for treatment by cochlear implantation, there is a need for greater understanding of the neurophysiological and neuroanatomical consequences of unilateral deafness, and more broadly, asymmetric hearing loss. In non-human primates with noise-induced asymmetric hearing loss, microelectrode-derived primary auditory cortical maps show plastic change in canonical microcircuits for the sound processing. That is, auditory cortex contralateral to the poorer ear undergoes time-dependent realignment of interaural frequency maps in association with elevated cortical thresholds to sound input from the better ear (Cheung et al., 2009). A follow-up study that contrast functional organization of the contralateral and ipsilateral hemispheres relative to the poorer ear demonstrates loss of normal mirror-image relationship across the two hemispheres, suggesting that whole brain alterations triggered by asymmetric hearing loss may account for impaired performance in tasks that require binaural input alignment or interhemispheric processing (Cheung et al., 2017). Parallel human brain imaging studies that aim to identify brain plasticity in SSD asymmetric hearing loss are limited. Magnetoencephalographic imaging (MEGI) studies that have used simple sound stimuli and examined low frequency phase-locked evoked activity in auditory cortices of adults with SSD. Interhemispheric differences in the M100 response provide evidence for temporal and spatial plasticity (Pross et al., 2015; Chang et al., 2016). Beyond auditory cortex, hearing restricted to one ear may have wider impact on functional networks that support attention and working memory demands during difficult listening tasks of complex acoustic stimuli. Cortical plasticity of such non-phase locked neural oscillatory activity across multiple frequency bands have to date not yet been examined in SSD.

In this study, we evaluate stimulus-induced neural oscillatory activity differences between adult onset SSD subjects with one NH ear against controls with two normal ears using a moderately difficult auditory syllable sequence reproduction task. We use MEGI to measure power fluctuations of non-phase locked activity across the whole brain for the entire spectrum of neural oscillations. Moreover, we use diffusion tensor imaging (DTI) to assess whole brain white matter structural integrity. We hypothesize that restriction of auditory input to one ear in adults with SSD results in both functional plasticity during accurate listening and structural plasticity of attention and working memory networks.

## Materials and Methods

### Subjects

This study protocol was approved by the Committee on Human Research at University of California, San Francisco (UCSF). Consent obtained from all subjects to participate in the study was both informed and written. Twenty-six adult onset SSD subjects [all values: mean ± standard deviation; age (years) = 48.7 ± 14.6; 12 female; one left handed] with unilateral hearing loss longer than 1 year duration were recruited from UCSF Otolaryngology and Audiology clinics. Of these 26 subjects, 15 were deaf in the left ear and 11 were deaf in the right ear. The average duration of deafness was 8.6 ± 8.7 years, ranging from 1 to 31 years. The average audiometric threshold of pure tone frequencies at 0.5, 1, 2, and 4 kHz for the deaf ear was 96.8 ± 18.4 dB HL, and the only hearing ear was 6.1 ± 5.8 dB HL. The etiology of sensorineural SSD was idiopathic in 18 subjects and acoustic neuroma in eight subjects that was managed by observation or gamma knife radiosurgery (no open craniotomy). Fifteen NH control subjects were recruited [age (years) = 46.6 ± 12.4; seven female; one left handed] from the UCSF community, matching age, gender, and handedness to the cohort of SSD patients (Table 1). The average audiometric threshold of pure tone frequencies at 0.5, 1, 2, and 4 kHz in control subjects was ≤25 dB HL in both ears. Exclusion criteria included implanted bone conduction devices, significant head trauma, seizure disorder, cerebrovascular accident, magnetic artifact, and contraindication to MRI. After data quality assessments and exclusions were applied, MEGI data was analyzable in 24 SSD patients and 14 NH controls and DTI data was analyzable in 20 SSD patients and 12 NH controls.

**Table 1:** Demographic data of SSD patients and normal hearing controls.

Cohorts	SSD	Control	*p*-Value^∗^
*N*	26	15	
Gender			
Male	14 (53.8%)	8 (53.3%)	0.975
Female	12 (46.2%)	7 (46.7%)	
Age (year)			
Mean (SD)	48.7 (14.6)	46.6 (12.4)	0.629
Range	22–77	25–61	
Handedness			
Right	25 (96.2%)	14 (93.3%)	0.604
Left	1 (3.8%)	1 (6.7%)	
Deaf ear			
Right	11 (42.3%)	N/A	
Left	15 (57.7%)	N/A	
Duration of deafness (year)			
Mean (SD)	8.6 (8.7)	N/A	
Range	1–31	N/A	
PTA threshold (dB HL)			
Good ear [mean (SD)]	6.1 (5.8)	N/A	
Deaf ear [mean (SD)]	96.8 (18.4)	N/A	
Etiology of deafness			
Acoustic neuroma	8 (30.8%)	N/A	
Idiopathic	18 (69.2%)	N/A	

*SSD, single-sided deafness; dB, decibel; HL, hearing level. ^∗^Statistical analyses for comparisons between groups were carried out using unpaired Student’s t, chi-square, and Fisher’s exact tests.*

### MEGI Acquisition and Task

Magnetoencephalographic imaging data were collected using a whole-head 275-channel CTF Omega 2000 biomagnetometer with third-order gradient correction (CTF Inc., Port Coquitlam, Canada), at 1200 Hz sampling rate. Radio-emitting coils were placed at the nasion and 1 cm anterior to the left and right preauricular points for head position tracking and co-registration with MRI (MPRAGE) data. MEGI sessions in which head movement exceeded 2 mm were discarded and repeated to maximize high data quality.

During MEGI, subjects performed an auditory syllable reproduction task (Herman et al., 2013). Subjects were instructed to listen to and vocally reproduce two- or four-syllable utterances presented binaurally during the imaging session. Stimuli were pre-recorded from a single female speaker and consisted of permutations of the syllables /ba/, /da/, and /pa/ (e.g., /ba da/ or /ba da pa ba/). At the beginning of each trial, subjects listened to the target presentation, waited for a visual “go” cue to respond (jittered between 2050 and 2150 ms after stimulus onset), and then vocally repeated back the stimulus pattern they heard. Syllables lasted 470 ms on average and were separated by 150 ms within a trial. After a go cue, subjects had up to 3 s to complete their response, after which the experiment proceeded to the next trial. Within two-syllable trials, no syllable was repeated, and within four-syllable trials, no syllable pair was repeated. Subjects had no previous knowledge or clues about the content of the upcoming trial. Eighty two- and four-syllable target trials were presented in pseudorandomized order. Correct and incorrect syllable repetition was recorded. Trials were labeled correct if the target syllables were repeated in proper order within 3 s of the go cue. Behavioral data (reaction time, accuracy) were analyzed using 2 × 2 ANOVA, by cohort (SSD, NH) and condition (two-syllable, four-syllable).

### MRI Acquisition

Structural MRI and DTI data were acquired from each subject on a 3-T MRI scanner (Discovery MR750, GE Medical system, Waukesha, WI, United States). Structural T1-weighted FSPGR BRAVO scans were obtained (120 axial slices, FOV = 512 mm × 512 mm, TR = 7232 ms, TE = 2.78 ms, in-plane voxel dimensions 0.5 mm × 0.5 mm, slice thickness = 1.5 mm). For the DTI protocol, a 12-min DTI (HARDI) scan was collected in 55 diffusion weighted directions (*b* = 3000 s/mm^2^, TR = 9313 ms, TE = 76.6 ms, FOV = 256 mm × 256 mm, in-plane voxel dimensions = 2 mm × 2 mm, axial slice thickness = 2 mm).

### MRI-DTI Analysis

Preprocessing of DTI data was performed with FSL (FMRIB Software Library, version 4.1.9, Oxford, United Kingdom) (Smith et al., 2004; Jenkinson et al., 2012), including correction for eddy current induced distortion and head motion. Diffusion-weighted images were registered to the non-diffusion weighted volume (*b* = 0) with six degrees of transformation (Smith, 2002). Fractional anisotropy (FA), mean diffusivity (MD), axial diffusivity (AD), and radial diffusivity (RD) maps were analyzed using tract-based spatial statistics (TBSS) in FSL (Smith et al., 2006). The diffusion tensor was estimated on a voxel-by-voxel basis using FSL’s DTIfit toolbox within the FMRIB Diffusion Toolbox of FSL (FSLv4.1.7^1^). The FA maps were then transformed in Montreal Neurological Institute (MNI) space to an anatomical template (FMRIB58_FA) using non-linear registration in FSL’s FNIRT. These parameters were then applied to the MD, AD, and RD maps for each subject.

A study-specific “skeleton,” representing the center of all fiber bundles common to all subjects was generated using an optimized FA threshold of 0.2 and confirmed by visual inspection. Each subject’s aligned FA, MD, AD, and RD maps were then projected onto this skeleton and entered into voxel-wise cross-subject statistics. Comparisons (independent *t*-tests) between groups (SSD and NH) were performed using permutation testing (5000 random permutations) applied to the general linear model implemented in FSL. Statistical maps were thresholded at *p* < 0.01 corrected for multiple comparisons at a cluster level using the TFCE (threshold-free cluster enhancement) approach. This threshold was relaxed in order to maximize conjunction with MEGI source reconstructions (see below), although significant group effects were still present at the *p* < 0.05 TFCE corrected level. Anatomical labels for white matter tracts were identified using the Johns Hopkins University (JHU) Atlas. To identify cortical structures impacted by white matter tract changes, we also used the Harvard-Oxford cortical atlas.

### MEGI Analysis

Magnetoencephalographic imaging data were epoched into -1 to 7 s trials relative to onset of the first syllable. Channels and trials with high frequency activity consistently >1.5 pT or in which the subject spoke during this interval were discarded. A forward model lead field describing the magnetic field strength at each sensor arising from a dipole source at each voxel was computed using a multiple local-spheres spherical volume conductor model (Dalal et al., 2008). All data were denoised using a novel algorithm, dual signal subspace projection (DSSP) for removing large interference and movement artifacts arising from speech production (Sekihara et al., 2016). Default choices were made for the spatial subspace dimension (*N* = 30) and temporal subspace dimension (*N* = 5). To avoid mislocalizations attributable to temporally correlated sources between the two hemispheres, data from sensors covering each hemisphere were analyzed separately (Dalal et al., 2008, 2011). The focus of our analysis was on induced neural oscillations during auditory stimulus encoding across the neocortical mantle. We evaluated all induced oscillations across all frequencies. After notch filtering ∼60 Hz, we segmented the data into four bands [4–8 Hz (θ), 8–13 Hz (α), 13–30 Hz (β), and 30–50 Hz (γ)] with a 60 and 120 Hz 1.5-Hz notch filter. Induced, phase-independent activity in each band was localized to the subject’s spatially normalized MRI using the NUTMEG time–frequency beam-forming spatially adaptive filter algorithm, which has been previously described in detail (Dalal et al., 2011). A time–frequency optimized beam-forming inverse solution for the dipole moment dependent on the lead field and sensor covariance was then computed for each voxel for each frequency band at every time window, averaged across overlapping time windows. Localizations were computed using the shared computing cluster at the California Institute for Quantitative Biomedical Research. ^2^ For activations, noise-corrected pseudo-*F* ratios were computed between active windows (i.e., stimulus) and a prestimulus baseline. Window sizes were frequency-band optimized (4–8 Hz: 400 ms; 8–13 Hz: 300 ms; 13–30 Hz: 200 ms; 30–50 Hz: 150 ms) with an overlap of 50 ms. The time windows centered from 75 to 475 ms after the beginning of stimulus were analyzed. Activations were computed from averaged single-trial data covariance for each time window and frequency band. Since we were primarily interested in induced oscillations during stimulus encoding, we grouped together two- and four-syllable trials to increase power of the response patterns.

For group analyses, tomographic volumes of potential dipolar source locations (voxels) were first generated in the subject’s native MRI and subsequently normalized to the template space. To enable neural-source localization, high-resolution anatomical MR images for each subject were spatially normalized to a standard MNI brain template using SPM2^3^ software. SnPM (statistical non-parametric mapping) unpaired *t*-tests were performed to compare the difference between SSD and NH cohorts with noise-corrected pseudo-*F* ratios (Dalal et al., 2008). In brief, time–frequency beam-formed images for each subject were first spatially normalized to the MNI template. The three-dimensional average and variance maps across subjects were calculated for each time–frequency window, and variance maps were smoothed with a 20 mm × 20 mm × 20 mm Gaussian kernel. From this image, a pseudo-*t* statistic was obtained at each voxel, time window, and frequency band. Non-parametric null distributions were created by permuting voxel labels (2*^N^* permutation, where *N* is the number of subjects) to derive *p*-values for the true image that were then corrected for multiple comparisons across all voxels, frequency bands, and time points using the cluster correction procedure. The threshold for significant changes was set to *k* > 20 whole brain cluster level with a cluster building threshold of *p* < 0.001 uncorrected at voxel level.

## Results

### Behavioral Performance

**FIGURE 1:**
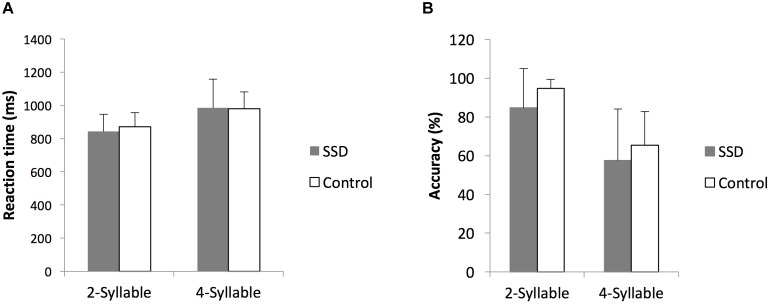
Comparison of reaction times and accuracy rates to two- and four-syllable phoneme tasks between single-sided deafness (SSD) and normal hearing control cohorts. **(A,B)** Reaction times and accuracy rates, respectively, are indistinguishable. ms, milliseconds; error bars, standard error of the mean.

In both cohorts, subject accuracy was higher (*p* < 0.05) and reaction times were faster (*p* < 0.05) for two-syllable trials compared to four-syllable trials, consistent with previous reports on this task (Herman et al., 2013). Comparing SSD and NH cohorts, there were no significant differences in performance on the two- or four-syllable repetition trials (Figure 1, all *p* > 0.05). Based on comparable psychoacoustic performance in the two cohorts, we interpret functional and structural imaging differences between SSD and NH as whole brain changes attributable to SSD not confounded by task performance differences.

### Neural Activation During Auditory Encoding

**FIGURE 2:**
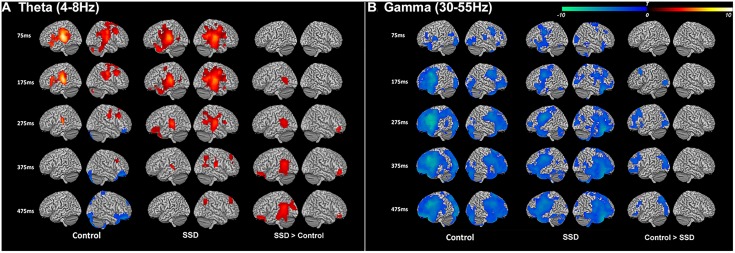
Induced theta band (4–8 Hz) and gamma band (30–50 Hz) neural oscillations during syllable encoding in normal hearing control and single-sided deafness (SSD) cohorts, and differences between the two cohorts. **(A)** Theta and **(B)** gamma band oscillations show similar recruitment of cortical regions in the two cohorts (left and middle columns). For within group contrasts, warm colors indicate increased magnitude relative to a pre-stimulus baseline in the theta band and cool colors indicate increased magnitude relative to a pre-stimulus baseline in the gamma band. **(A)** SSD cohort has increased recruitment (right column) of higher order auditory cortex in the theta band. **(B)** SSD cohort has decreased recruitment attention and working memory networks (frontal, parietal, and occipital cortical regions) in the gamma band. In SSD and control cohort contrasts, warm colors in the theta band indicate regions where activity is increased in SSD and cool colors in the gamma band indicate regions where activity is decreased in SSD. ms, milliseconds.

We first reconstructed induced oscillatory neural activity during the stimulus period, computing averages with respect to a pre-stimulus baseline for SSD patients and NH controls (Figure 2). To illustrate that the two cohorts share the same activation pattern, we reconstructed time–frequency domain neural activity patterns for each cohort, 14 SSD patients and 14 NH controls, pairwise matched for age, gender, and handedness. This within group analysis revealed that the two cohorts displayed qualitatively similar activation patterns across all frequency bands. During stimulus encoding, induced power changes were positive in the theta and alpha bands from 50 to 200 ms post-stimulus, but were negative in the beta and gamma bands from 150 to 500 ms post-stimulus. However, the across group contrast of 24 SSD patients and 14 NH controls revealed significant quantitative differences in induced oscillatory activity in the theta (4–8 Hz) and gamma (30–55 Hz) bands, mainly over the left hemisphere (Figure 2 and Table 2). Specifically, between 175 and 475 ms following stimulus onset, the SSD cohort showed increased induced theta band oscillations in the left superior temporal cortex and decreased induced gamma band oscillations in the left frontal, superior parietal, and occipital cortices (Figure 2 and Table 2).

**Table 2:** Tabulation of all brain regions showing significant differences between SSD and control cohorts that include a cluster size >20 voxels and uncorrected *p*-value <0.001.

Band (Hz)	Duration (ms)	Peak (ms)	Anatomic region	BA	*x*	*y*	*z*	*T*
4–8	125–475	475	Left superior temporal gyrus	13	−40	−50	20	−4.1
4–8	275–475	375	Right middle frontal gyrus	11	30	45	−10	−3.62
4–8	275–475	475	Right precuneus	7	15	−55	40	−3.95
4–8	325–475	425	Left rectal gyrus	11	0	40	−25	−3.56
4–8	425–475	475	Left cuneus	18	−20	−85	15	−3.27
30–55	175–375	375	Left superior frontal gyrus	8	−10	25	50	−3.9
30–55	175–475	375	Left middle occipital gyrus	19	−25	−85	15	−3.76
30–55	225–375	325	Left middle frontal gyrus	10	−35	40	30	−3.88
30–55	225–375	325	Left middle frontal gyrus	11	−40	40	−15	−3.61
30–55	275–325	325	Left precentral gyrus	44	−65	10	5	−3.63
30–55	275–475	375	Left superior occipital gyrus	19	−45	−85	30	−4.96

*Frequency band, time windows of activation differences relative to stimulus onset, peak latency of differential activation between cohorts, the anatomic region label with corresponding Brodmann Area (BA), Montreal Neurological Institute coordinates (x, y, and z), and T values are listed.*

### White Matter Structural Integrity

**FIGURE 3:**
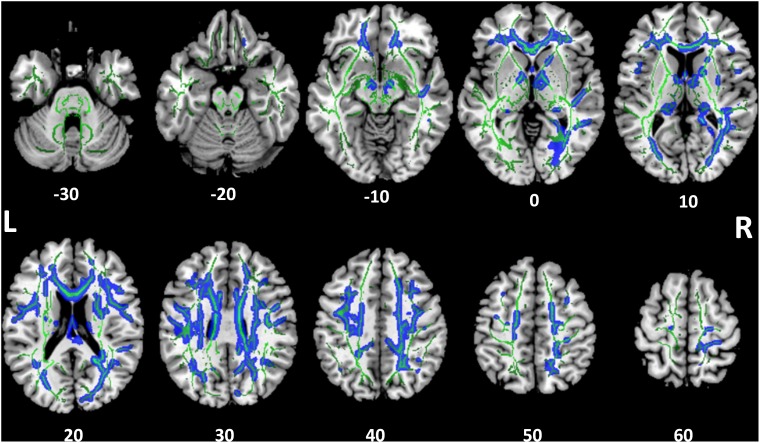
Tract-based spatial statistics for fractional anisotropy (FA). Widespread tracts with decreased FA in SSD patients compared to control subjects (blue) are superimposed on the skeleton (green) of tracts common to both cohorts. The *z* coordinate in the Montreal Neurological Institute (MNI) space for each axial section is noted at bottom. L, left; R, right.

**FIGURE 4:**
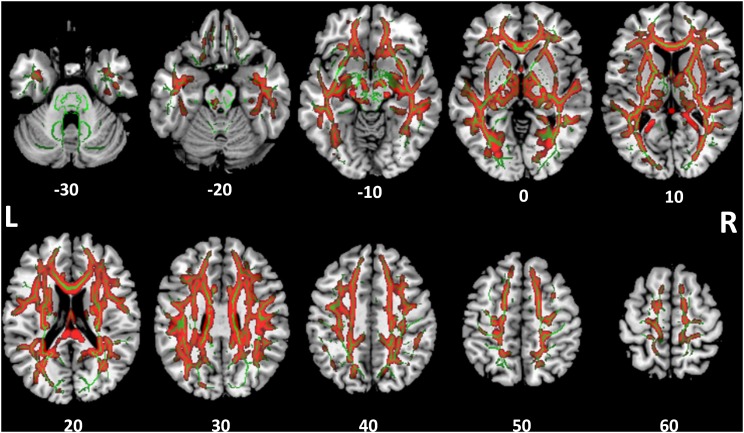
Tract-based spatial statistics for medial diffusivity (MD). Widespread tracts with increased MD in SSD patients compared to control subjects (red) are superimposed on the skeleton (green) of tracts common to both cohorts. The *z* coordinate in the MNI space for each axial section is noted at bottom.

**FIGURE 5:**
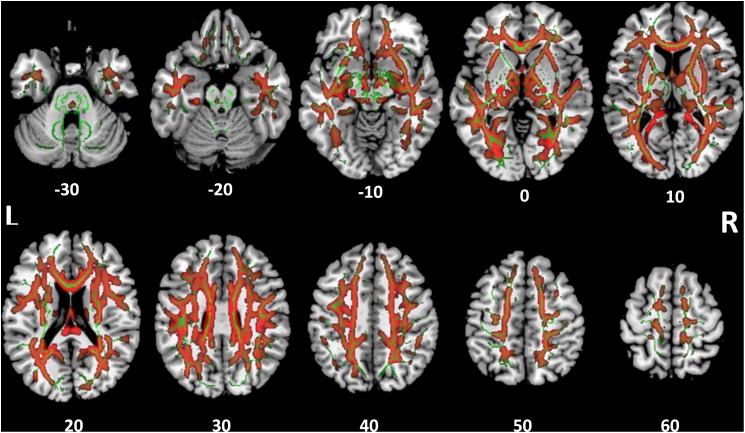
Tract-based spatial statistics for radial diffusivity (RD). Widespread tracts with increased RD in SSD patients compared to control subjects (red) are superimposed on the skeleton (green) of tracts common to both cohorts. The *z* coordinate in the MNI space for each axial section is noted at bottom.

Tract-based spatial statistics analysis showed the SSD cohort to have significantly reduced FA for multiple white matter skeleton clusters (Figure 3) and increased MD and RD for more extensive tracts that overlapped with the region of reduced FA (Figures 4, 5). By contrast, there were only modest increases in AD that were restricted to a very limited area (data not shown). When mapped to the JHU white matter atlas, FA reduction was observed in the genu, body and splenium of the corpus callosum, fornix, bilateral anterior, superior and posterior corona radiata, anterior and posterior limb of internal capsule, posterior thalamic radiation (including optic radiation), fornix (cres)/stria terminalis, superior longitudinal fasciculus, cingulum (cingulate gyrus), and right superior fronto-occipital fasciculus and external capsule (Table 3). When mapped to the Harvard-Oxford cortical atlas, FA reduction was identified in the frontal pole, insular cortex, superior, middle and inferior frontal gyruses, precentral gyrus, posterior division of superior temporal gyrus, temporo-occipital part of middle and inferior temporal gyrus, postcentral gyrus, superior parietal lobule, supramarginal gyrus, angular gyrus, lateral occipital cortex, intracalcarine cortex, frontal medial cortex, subcallosal cortex, paracingulate gyrus, cingulate gyrus, precuneus cortex, cuneal cortex, frontal orbital cortex, lingual gyrus, occipital fusiform gyrus, frontal operculum cortex, central operculum cortex, planum polare, Heschl’s gyrus, planum temporale, supracalcarine cortex, and occipital pole (Table 3). The SSD cohort showed reduction in FA across many regions, indicating deafness in one ear broadly impacted white matter structural integrity throughout the brain.

**Table 3:** White matter regions with decreased FA in SSD patients compared to the normal hearing controls.

Anatomic regions	Voxels	Anatomic regions	Voxels	Anatomic regions	Voxels
**Johns Hopkins University White Matter Atlas**					
Genu of corpus callosum	884	Anterior corona radiata R	551	Cingulum (cingulated gyrus) R	296
Body of corpus callosum	2565	Anterior corona radiata L	889	Cingulum (cingulated gyrus) L	184
Splenium of corpus callosum	287	Superior corona radiata R	1087	Fornix (cres)/stria terminalis R	39
Fornix	104	Superior corona radiata L	723	Fornix (cres)/stria terminalis L	39
Anterior limb of internal capsule R	304	Posterior corona radiata R	495	Superior longitudinal fasciculus R	399
Anterior limb of internal capsule L	51	Posterior corona radiata L	120	Superior longitudinal fasciculus L	489
Posterior limb of internal capsule R	54	Posterior thalamic radiata (include optic radiation) R	453	Superior fronto-occipital fasciculus R	55
Posterior limb of internal capsule L	49	Posterior thalamic radiata (include optic radiation) L	73		
Retrolenticular part of internal capsule R	22	External capsule R	59		
**Harvard-Oxford Cortical Structural Atlas**					
Frontal pole	529	Supramarginal gyrus, anterior division	43	Cuneal cortex	126
Insular cortex	68	Supramarginal gyrus, posterior division	200	Frontal orbital cortex	237
Superior frontal gyrus	508	Angular gyrus	400	Lingual gyrus	100
Middle frontal gyrus	1303	Lateral occipital cortex, superior division	1124	Occipital fusiform gyrus	77
Inferior frontal gyrus, pars triangularis	412	Lateral occipital cortex, inferior division	78	Frontal operculum cortex	77
Inferior frontal gyrus, pars opercularis	318	Intracalcarine cortex	310	Central operculum cortex	140
Precentral gyrus	1953	Frontal medial cortex	161	Planum polare	144
Superior temporal gyrus, posterior division	79	Subcallosal cortex	177	Heschl’s gyrus	99
Middle temporal gyrus, temporooccipital part	281	Paracingulate gyrus	100	Planum temporale	210
Inferior temporal gyrus, temporooccipital part	18	Cingulate gyrus, anterior division	1844	Supracalcarine cortex	88
Postcentral gyrus	1101	Cingulate gyrus, posterior division	978	Occipital pole	49
Superior parietal lobule	242	Precuneus cortex	944		

*The regions are determined in accordance to the Johns Hopkins University and Oxford-Harvard atlases. FA, fractional anisotropy; SSD, single-sided deafness; R, right hemisphere; L, left hemisphere.*

### Overlay of MEGI and DTI Maps

**FIGURE 6:**
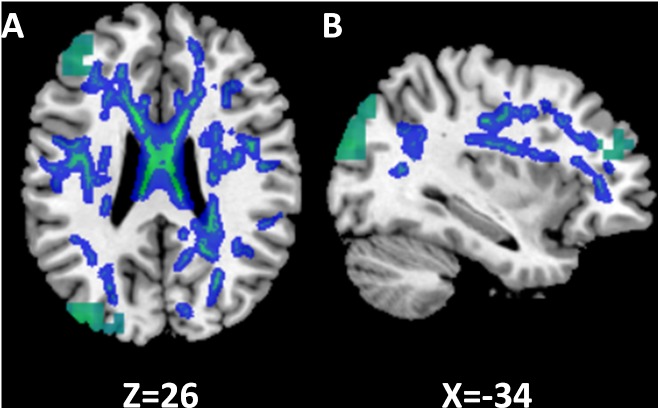
Relationship between functional and structural differences in single-sided deafness (SSD). In the SSD cohort, tracts with decreased FA are subjacent to cortical regions with decreased activation during performance of the auditory working memory task (gamma band, time window 375 ms). The *z* and *x* coordinates in the MNI space are shown at the bottom of axial and sagittal images. **(A)** Axial. **(B)** Sagittal.

To examine functional changes in the context of white matter structural integrity in SSD, we combined gamma band findings on MEGI with white matter results from DTI by generating an overlay of these two statistical maps (Figure 6). In the SSD cohort, there were two regions where reductions in gamma (30–55 Hz) power (Figure 6, green) overlapped with reductions in white matter integrity (Figure 6, blue). First, the cluster of reduced gamma power over the left frontal pole was adjacent to reduced FA in the superior longitudinal fasciculus. Second, the region of reduced gamma power over the superior division of parietal cortex on MEGI was also adjacent to the superior longitudinal fasciculus. The SSD cohort had reduced gamma band oscillatory power over the frontal and parietal cortices that were accompanied by impaired integrity of related white matter tracts. Long-term obligatory monaural hearing impacted cortical induced activity and subcortical white matter microstructure of the dorsal attention network (frontal and parietal regions), providing evidence for co-occurrence of functional and structural plasticity in SSD.

## Discussion

In a first of its kind adult SSD multimodal structure-function imaging study, we measured cortical activity during an auditory stimulus encoding task on millisecond time and sub-centimeter spatial resolution scales and assessed whole brain white matter structural integrity. We identified functional plasticity in SSD, manifested by enhanced activation of higher order auditory cortices in the left hemisphere, suggesting hyperactivity to support comparable performance accuracy relative to NH in a difficult listening task. We also observed corresponding reductions in task-related induced gamma band oscillatory activity in the left frontal and parietal cortices, brain regions implicated in supporting attention and working memory (Rauschecker and Scott, 2009; Rauschecker, 2012, 2018), findings consistent with disengagement of these cortical networks in SSD. Documented functional changes were associated with structural changes in white matter tracts neighboring these regions.

Individuals afflicted by SSD often face considerable auditory processing demands posed by rapid sequential or overlapping stimuli in real-life communicative interactions. We assessed the neural correlates of monaural hearing under cognitive duress by choosing a demanding auditory syllable encoding task that challenged working memory. We established comparable reaction time and accuracy performance in the two cohorts to remove any confounds introduced by unequal hearing competencies. Therefore, our study design directly queried changes in oscillatory network activity due to unilateral deafness.

In earlier adult SSD studies, we found evidence for functional plasticity of early sensory cortices using MEGI. Auditory cortical responses to simple tones revealed spatial plasticity, expressed as increased activation spread distance in the hemisphere contralateral to the only hearing ear and decreased distance in the ipsilateral hemisphere (Chang et al., 2016) and temporal plasticity, expressed as loss of normal interhemispheric latency difference of the M100 signal (Pross et al., 2015). In the current study, the SSD cohort expressed enhanced theta-band oscillations of higher order auditory cortex during encoding of multisyllabic sequences, showing increased recruitment for accurate listening performance and another facet of within-modality sensory cortical plasticity.

Our MEGI observation of task-related gamma band activity in frontal, parietal, and occipital regions is consistent with published accounts of electrocorticography recordings during a working memory task that show recruitment of these regions (Jerbi et al., 2010; Ossandon et al., 2011, 2012; Ramot et al., 2012). Attention and working memory demands of the syllable sequence reproduction task activated these brain networks in both cohorts. However, the SSD cohort expressed reduced gamma band activity in those networks, a broader manifestation of cortical plasticity revealed by the phoneme replication task. This finding suggests that chronically overtaxed cognitive subsystems in SSD can no longer allocate comparable resources relative to NH subjects to perform the challenging auditory task. Taken together, decreased recruitment of overburdened attention and working memory network along with increased recruitment of higher order auditory cortices may be neuroimaging biomarkers for impairment of ease of communication in SSD (Rigby et al., 1997; Gatehouse and Noble, 2004).

Another form of cortical plasticity, namely, cross-modality audiovisual, has been found in few task-based and resting-state fMRI studies. In children with SSD, lower activation of secondary visual processing regions on an audiovisual task has been reported (Schmithorst et al., 2014). In adults with SSD, resting-state fMRI reveals decreased functional connectivity between primary auditory cortex and visual cortex (Liu et al., 2015) and enhanced synchronous output entropy connectivity between left primary auditory cortex with certain regions of visual networks (Zhang et al., 2016). The current study did not explicitly explore audiovisual integration, and our data cannot exclude the possibility of such plasticity expressed as change in induced neural oscillation.

We also evaluated whether the observed functional modifications may be accompanied by structural changes. There were widespread reductions of white matter integrity in multiple tracts, including those that connect regions of altered activity as revealed by MEGI. First, FA reduction was extensive and observed not only in auditory tracts but also in multiple non-auditory tracts. Second, tracts with FA reduction were mainly located in the dorsal cortical regions. It has been well accepted that there are ventral and dorsal processing streams in auditory cortex, responsible for the perception of “what” and “where” aspects of sound processing (Rauschecker and Tian, 2000; Hickok and Poeppel, 2004; Rauschecker and Scott, 2009). We surmise that the dorsal auditory pathways are more likely impacted in SSD. Furthermore, with combined functional and structural alterations revealed by a challenging auditory working memory task, immediate follow up questions on the causal relationships and sequence of events between structural and functional brain plasticity in SSD will require additional studies.

Whereas several studies have used DTI to investigate structural changes in SSD, they focused mainly on auditory pathways; much less attention was devoted to non-auditory pathways (Wu et al., 2009; Rachakonda et al., 2014; Vos et al., 2015). We deployed whole brain TBSS analyses without predefining voxels or tracts of interest (Smith et al., 2006). To the best of our knowledge, no SSD study has used this approach for DTI analyses. Our analyses revealed the SSD cohort had significant reductions of FA, driven by increased MD and RD but not AD in several non-auditory tracts, implicating axonal demyelination as opposed to fiber loss as the main mechanism of structural change. Other SSD studies in the literature also report decreased FA, with inconsistent effects on other diffusion parameters (Lin et al., 2008; Wu et al., 2009; Miao et al., 2013; Hribar et al., 2014). Differences between the present study and prior studies may be due to variations in study populations, demographic features, onset of deafness, and congenital versus acquired deafness.

We recognize important limitations to this study. The number of subjects in both SSD and NH cohorts were relatively modest, resulting in reduced sensitivity to make group level inferences that may be more significant for diffusion imaging data. And, inadequate number of subjects to address right versus left deafness in the SSD cohort necessitated analysis of the entire SSD cohort without differentiation of deafness laterality to achieve suitable statistical power. The literature indicates that aspects brain plasticity in SSD may be dependent on side of deafness (Schmithorst et al., 2014; Zhang et al., 2016). Furthermore, covariates of duration of deafness, age of deafness onset, labyrinthine dysfunction, namely tinnitus and vertigo, were not explicitly studied. Those clinical features may also alter functional and structural manifestations of brain plasticity (Schmidt et al., 2013; Benson et al., 2014; Seydell-Greenwald et al., 2014; Hinkley et al., 2015; Ryu et al., 2015). Future investigations should include much larger cohorts with more detailed clinical assessments to address both issues.

## Author Contributions

SN, PM, and SC contributed to the conception and design of the study. CG and DM organized the database. YS, LH, CC, KS, Y-SC, and JO performed the statistical analysis. YS wrote the first draft of the manuscript. SN and SC wrote sections of the manuscript. All authors contributed to manuscript revision, read, and approved the submitted version.

## Conflict of Interest Statement

The authors declare that the research was conducted in the absence of any commercial or financial relationships that could be construed as a potential conflict of interest.

## Footnotes

1 https://www.fmrib.ox.ac.uk/fsl

2 www.qb3.org

3 http://www.fil.ion.ucl.ac.uk/spm/software/spm2/
